# Diagnosis and underdiagnosis of comorbidities in psoriasis patients - need for a multidisciplinary approach*

**DOI:** 10.1590/abd1806-4841.20164716

**Published:** 2016

**Authors:** Gleison Vieira Duarte, Maria de Fátima S. P. de Oliveira, Ivonise Follador, Thadeu Santo Silva, Edgar Marcelino de Carvalho Filho

**Affiliations:** 1 Instituto Bahiano de Imunoterapia (IBIS) – Salvador (BA), Brazil; 2 Universidade Federal da Bahia (UFBA) – Salvador (BA), Brazil

**Keywords:** Anthropometry, Clinical diagnosis, Epidemiologic factors, Metabolic syndrome X, Prognosis, Psoriasis

## Abstract

**BACKGROUND:**

Psoriasis is an immune-mediated disease that manifests predominantly in the skin, although systemic involvement may also occur. Although associated comorbidities have long been recognized and despite several studies indicating psoriasis as an independent risk factor for cardiovascular events, little has been done in general medical practice regardind screening. In the United States, less than 50% of clinicians are aware of these recommendations.

**OBJECTIVE:**

To identify the prevalence of these comorbidities in 296 patients followed up at a university dermatology clinic.

**METHODS:**

Systematically investigated comorbidity frequencies were compared with general practitioners' registry frequencies. Clinical features correlated with comorbidities were also investigated.

**RESULTS:**

High prevalences of systematically investigated comorbidities such as hypertension (30%) and dyslipidemia (26.5%) were documented. Conversely, data from general practitioners' records showed that 33% of dyslipidemia cases were undiagnosed and indicated possible underdiagnosis of some comorbidities. Furthermore, an association was found between: the number of comorbidities and psoriasis duration, age and high body mass index an association was found between the number of comorbidities and psoriasis duration, age, high body mass index, waist circumference or waist-to-hip ratio. (p<0.05).

**CONCLUSION:**

Disease duration, age and high body mass index, waist circumference or waist-to-hip ratio are possible criteria for choosing which patients should be screened for comorbidities. Underdiagnosis of comorbidities by general practitioners highlights the need for a multidisciplinary approach in psoriasis management.

## INTRODUCTION

Since 1818, when an association between psoriasis and joint disorders (psoriatic arthritis) was reported, various other comorbidities have been described, including systemic arterial hypertension (SAH), diabetes mellitus (DM), and cardiovascular disease.^1,2^ Other comorbidities, such as celiac disease and erectile dysfunction, have been described more recently.^3-5^ The clinical manifestations of comorbidities generally appear years after the onset of psoriasis and are more common in severe cases.^6-8^ Furthermore, a high frequency of smoking has been noted in psoriasis patients.^9^ Systematic reviews have consistently demonstrated an increased cardiovascular risk and the need for appropriate prevention measures.^10-12^

Alarming rates of obesity and overweight were described in Brazilian psoriasis patients. Moreover, waist circumference and waist-to-hip ratio (WHR) showed a better correlation with the psoriasis severity.^13^

Although associated comorbidities have long been recognized, there may be a theory-practice dissociation in psoriasis care by dermatologists and there remains an unanswered question over how comorbidities should be screened and who should undertake this task.

This study aimed to determine the prevalences of SAH, DM, dyslipidemia (DLP) and smoking consumption in psoriasis patients (by screening every patient actively), and ascertain the prevalence of other previously diagnosed comorbidities (data from general practitioners' (GPs) registries). In addition, the features bearing a high correlation with comorbidities were investigated.

## METHODS

The data analyzed in the present cross-sectional study were obtained from a sample of 296 psoriasis patients followed up at the Dermatology Service of the Federal University of Bahia, between October 1, 2008 and August 10, 2010. The primary objective of this early study was to evaluate the prevalence of obesity.^13^ The study was approved by the institution's Ethics Committee. The sample size calculation was based on the formula n =z · p · q/d^2^. It took into account a prevalence ("p") of obesity in the state of Bahia, Brazil, of 5%, according to data sourced from the Brazilian Ministry of Health, and given a variation ("d") of ±5%. A structured questionnaire was used for data collection. Every psoriasis patient aged ≥ 18 was invited and all consenting consecutive individuals were enrolled. Exclusions motivated by unwillingness to participate in the study were not registered.

### Specific procedures:

The psoriasis area severity index (PASI) was used to assess psoriasis severity and a score of ≥ 10 was considered moderate-to-severe psoriasis.^14^Anthropometric evaluations included BMI, waist circumference and WHR.

Diagnoses of SAH and DM were made in accordance with the guidelines established by the American Heart Association. A diagnosis of DLP was made in the presence of two repeated measurements, showing the following levels: triglycerides ≥150 mg/dl, or high-density lipoprotein cholesterol (HDL-C) ≤ 40 mg/dl in men or ≤ 50 mg/dl in women, or low-density lipoprotein cholesterol (LDL-C) ≥130 mg/dl.^15^

Smoking was evaluated in pack-years, both for current and former smokers. Current smokers were also stratified in accordance to the mean number of cigarettes smoked/day: < 10 cigarettes/day, 10-20 cigarettes/day or > 20 cigarettes/day.^16^

Other comorbidities were obtained by examining the patients' GP medical records. Criteria and investigations used for their detection are not thoroughly described here (retrospective data).

### Data analysis

Statistical analysis was conducted using the SPSS version 11.5 for Windows. The continuous variables were described as means ± standard deviation of the mean, and the categorical variables as proportions. The distribution of the variables was tested for normality using the Kolmogorov-Smirnov test. Analyses of continuous variables were performed through the Kruskal-Wallis non-parametric test or Student's t-test, in accordance to the normality of distribution. Furthermore, analyses of association between the number of comorbidities and the categorical variables were conducted via the chi-square test. A significance level of 5% was set.

## RESULTS

A total of 296 psoriasis patients were included, 164 (55.4%) of whom were males. The mean age was 48 ± 14 years and the mean PASI score was 7.9 ± 7.7 (range 0 – 48). Severe psoriasis was significantly more common in men (37.8%) than in women (26.5%) (p = 0.04). Overall, 28.6% of the patients were obese.^13^

The frequencies of comorbidities are shown in table 1. Excluding obesity and psoriatic arthritis, 85 patients (32%) had one of the comorbidities listed in table 1, 32 (10.8%) had two and 25 (8.5%) had three or more comorbidities. Repeat measurements of arterial pressure, fasting glucose and lipid profile revealed no further SAH cases, though 2 more DM cases and 19 more DLP cases were uncovered, in addition to those previously diagnosed by GPs. Consequently, the frequency of DLP increased from 19.5%, in medical records, to 26%.

**Table 1:** The principal comorbidities registered in the study population

Comorbidity	Absolut frequency	Prevalence
DM	28*	9.0
DLP	57*	19.5
SAH	88*	30
**Hepatopathy (and NAFLD)**	5	1.7
Human immunodeficiency virus (HIV)	1	.3
Hepatitis C – clinical hepatitis or serology	2	.7
Hepatitis B – clinical hepatitis or serology	1	.3
**Stroke**	1	.3
**Coronary artery disease**	7	2.7
Peripheral vascular disease	5	1.7
Chagas disease	3	1.0
**Depression**	8	2.7
Diabetic retinopathy	1	.3
Bipolar disorder	1	.3
**Osteoarthrosis**	2	.7
Congestive heart failure	1	.3
Unspecified cancer	3	1.0
Vitiligo	2	.7

* No cases of hypertension, a further 2 cases of DM and 19 cases of DLP were identified following specific screening.

Possible cases of underdiagnosis are marked in bold. A different diagnosis could occur in the same patient. DM – diabetes; DLP – dyslipidemia; SAH- hypertension; NAFLD – non-alcoholic fatty liver disease.

When the variables sex, nail involvement, facial involvement, psoriatic arthritis, PASI score > 10, mean PASI score, and family history of psoriasis were analyzed, no association was found between any of the variables and the number of comorbidities (p>0.05). Statistical significance was found for the association between mean age, mean disease duration, higher BMI, WHR, waist circumference and the number of comorbidities (0, 1, 2 or ≥ 3 comorbidities) (Table 2).

**Table 2:** Comparison of subgroups of patients according to the number of comorbidities

Variable	Number of comorbidities				p-value
	0	1	2	≥3	
N	143 (48.5%)	95 (32.2%)	32 (10.8%)	25 (8.5%)	
Male	80 (27.1%)	52 (17.6%)	17 (5.8%)	15 (5.1%)	**c2 test**
					**p=0.958**
Mean age (n, SD)	43.1± 15.5	51.1 ± 11.5	54.5 ± 12.5	58.1 ± 11.4	**F Test**
					**p<0.00**
Time of psoriasis (months)	116 ± 96	152 ± 119	187 ± 156	159 ± 115	**K-W Test**
					**p=0.015**
Nail involvement	56 (19.0%)	43 (14.6%)	14 (4.7%)	9 (3.1%)	**c2 test**
					**p=0.740**
Facial involvement	86 (29.4%)	59 (20.1%)	20 (6.8%)	15 (5.1%)	**c2 test**
					**p=0.978**
Psoriatic arthritis	20 (6.8%)	17 (5.8%)	4 (1.4%)	5 (1.7%)	**c2 test**
					**p=0.740**
Inverted psoriasis	1 (3.0%)	1 (3.0%)	2 (0.7%)	3 (1.0%)	**c2 test**
					**p=0.002**
PASI score > 10	50 (16.9%)	32 (10.8%)	7 (2.4%)	8 (2.7%)	**c2 test**
					**p=0.557**
Family history of psoriasis	30 (10.3%)	21 (7.2%)	12 (4.1%)	8 (2.8%)	**c2 test**
					**p=0.093**
PASI score	8.6	8.1	7.0	7.1	**K-W test**
					**p=0.689**
BMI	26.7	28.7	29.5	30.5	**K-W test**
					**p=0.000**
Waist-to-hip ratio	0.90	0.94	0.96	0.98	**K-W test**
					**p=0.000**
Waist circumference (cm)	89.7	96.7	100.4	101.6	**K-W test**
					**p=0.000**

Statistically significant differences are marked in bold (Chi-square test; Anova; and Kruskal Wallis test). N=295 (waist circumference missing value for one patient). BMI – body mass index; PASI – psoriasis area severity index.

In this study sample, 42.9% of patients reported current or past smoking (14 ± 20 pack-years). There was no statistically significant difference in PASI scores between current/past smokers (n=116) and never-smokers (n=180) (p=0.79). In current smokers, comparison of PASI scores in groups stratified in accordance with daily cigarette consumption revealed no statistically significant differences (p>0.05).

## DISCUSSION

Our study confirms that comorbidities are associated with increased age, psoriasis duration, BMI, WHR and waist circumference. According to the literature, the association between cardiovascular risk factors and psoriasis remains even after adjustments for sex and age.^17^ Additionally, it was noted that male patients were more likely to have more severe forms of psoriasis, as previously reported, but neither gender nor psoriasis severity were associated with the occurrence of comorbidities.^18,19^ In contrast, however, previous studies have demonstrated that the greater the psoriasis severity, the stronger the association with comorbidities and, consequently, the higher the patient's cardiovascular risk.^20-22^

During the follow-up, 19 new DLP cases and 2 new SAH cases were identified, suggesting insufficient screening by GPs. The frequencies found for SAH and DM in this outpatient sample were similar to those found in other studies.^11,17^

DM incidence in patients with psoriasis correlates positively with BMI, the duration of the disease and/or the use of systemic therapy. ^23^ DLP has consistently been associated with psoriasis even after adjustment for other known, confounding factors. ^24-26^ In this study, DLP active screening increased diagnosis in 33% (19/57) of patients. Despite the impact of retinoids, cyclosporine and anti-TNF-α agents on lipid levels, the authors believe that the majority of undiagnosed cases is due to increased medical surveillance. ^27-29^

Epidemiological data from the Brazilian population show the following frequencies: SAH (33.7%); DM (8.0%); smoking (25.2%) and DLP (24.2%).^30-32^ Notably, the frequencies for several of the comorbidities evaluated were lower than the rates reported in the literature. For example, the rate of depression – also associated with an increased risk of cardiovascular disease – was almost ten times less than the expected.^33^ This underdiagnosis merits the utmost attention, since in up to 10% of psoriasis cases, it is severe enough to induce suicidal ideation.^34^ A similar level of underdiagnosis (1.7%) was found for non-alcoholic fatty liver disease, much lower than the level reported in the literature.^27-29^

Despite the publication of several population-based studies indicating psoriasis as an independent risk factor for cardiovascular events, motivating the adoption of screening for additional risk factors, it is known that these guidelines are largely ignored. ^35,36^ In the United States, less than 50% of clinicians are aware of these recommendations.^37^ In Europe, 27.7% and 44.2% of psoriatic patients with SAH and DLP respectively received no antihypertensive and cholesterol-lowering pharmacotherapy.^38^

Attempts to associate PASI score with smoking have yielded contradictory results.^9,16^ No correlation was uncovered between PASI and smoking. Current non-smoker patients might be affected by past smoking, as the effect of smoking is only nulled after 20 years of abstinence, making it difficult to isolate the influence of chronic nicotine abuse on psoriasis severity.^39,40^

This research has some limitations, including sample size and impossibility to establish causal relationships between correlated variables.

## CONCLUSION

This study shows that, despite being known for decades, some comorbidities are underdiagnosed in psoriasis patients. This finding serves as an alert to clinicians, dermatologists and healthcare authorities, emphasizing the need for multidisciplinary management and active screening, which would reduce the impact of these comorbidity associations on patients' survival, quality of life and mortality, reinforcing the need for changes in healthcare in concordance with growing evidence of theory-practice dissociation in psoriasis management.

The data from this and a previously published study^13^ enhance our understanding of increases in comorbidities according to age, time of psoriasis, BMI, WHR and waist circumference, which are possible criteria for determining which patients should be screened for comorbidities.

## References

[1] Henseler T, Christophers E (1995). Disease concomitance in psoriasis. J Am Acad Dermatol.

[2] Christophers E (2007). Comorbidities in psoriasis. Clin Dermatol.

[3] Birkenfeld S, Dreiher J, Weitzman D, Cohen AD (2009). Coeliac disease associated with psoriasis. Br J Dermatol.

[4] Gisondi P, Del Giglio M, Cozzi A, Girolomoni G (2010). Psoriasis, the liver, and the gastrointestinal
tract. Dermatol Ther.

[5] Goulding JM, Price CL, Defty CL, Hulangamuwa CS, Bader E, Ahmed I (2011). Erectile dysfunction in patients with psoriasis increased
prevalence, an unmet need, and a chance to intervene. Br J Dermatol.

[6] Naldi L, Mercuri SR (2010). Epidemiology of comorbidities in psoriasis. Dermatol Ther.

[7] Kimball AB, Gladman D, Gelfand JM, Gordon K, Horn EJ, Korman NJ (2008). National Psoriasis Foundation clinical consensus on psoriasis
comorbidities and recommendations for screening. J Am Acad Dermatol.

[8] Chang YT, Chen TJ, Liu PC, Chen YC, Chen YJ, Huang YL (2009). Epidemiological study of psoriasis in the national health
insurance database in Taiwan. Acta Derm Venereol.

[9] Wolk K, Mallbris L, Larsson P, Rosenblad A, Vingård E, Ståhle M (2009). Excessive body weight and smoking associates with a high risk of
onset of plaque psoriasis. Acta Derm Venereol.

[10] Hugh J, Van Voorhees AS, Nijhawan RI, Bagel J, Lebwohl M, Blauvelt A (2014). From the Medical Board of the National Psoriasis Foundation The
risk of cardiovascular disease in individuals with psoriasis and the
potential impact of current therapies. J Am Acad Dermatol.

[11] Horreau C, Pouplard C, Brenaut E, Barnetche T, Misery L, Cribier B (2013). Cardiovascular morbidity and mortality in psoriasis and psoriatic
arthritis a systematic literature review. J Eur Acad Dermatol Venereol.

[12] Richard MA, Barnetche T, Horreau C, Brenaut E, Pouplard C, Aractingi S (2013). Psoriasis, cardiovascular events, cancer risk and alcohol use
evidence-based recommendations based on systematic review and expert
opinion. J Eur Acad Dermatol Venereol.

[13] Duarte GV, Oliveira Mde F, Cardoso TM, Follador I, Silva TS, Cavalheiro CM (2013). Association between obesity measured by different parameters and
severity of psoriasis. Int J Dermatol.

[14] Finlay (2005). Current severe psoriasis and the Rule of Tens. Br J Dermatol.

[15] Lloyd-Jones DM, Hong Y, Labarthe D, Mozaffarian D, Appel LJ, Van Horn L (2010). Defining and setting national goals for cardiovascular health
promotion and disease reduction the American Heart Association's strategic
Impact Goal through 2020 and beyond. Circulation.

[16] Fortes C, Mastroeni S, Leffondré K, Sampogna F, Melchi F, Mazzotti E (2005). Relationship between smoking and the clinical severity of
psoriasis. Arch Dermatol.

[17] Neimann AL, Shin DB, Wang X, Margolis DJ, Troxel AB, Gelfand JM (2006). Prevalence of cardiovascular risk factors in patients with
psoriasis. J Am Acad Dermatol.

[18] Neimann AL, Shin DB, Wang X, Margolis DJ, Troxel AB, Gelfand JM (2006). Relationships between obesity and the clinical severity of
psoriasis in Taiwan. J Am Acad Dermatol.

[19] Reich K, Ortonne JP, Kerkmann U, Wang Y, Saurat JH, Papp K (2010). Skin and nail responses after 1 year of infliximab therapy in
patients with moderate-to-severe psoriasis a retrospective analysis of the
EXPRESS Trial. Dermatology.

[20] Augustin M, Reich K, Glaeske G, Schaefer I, Radtke M (2010). Co-morbidity and age-related prevalence of psoriasis analysis of
health insurance data in Germany. Acta Derm Venereol.

[21] Langan SM, Seminara NM, Shin DB, Troxel AB, Kimmel SE, Mehta NN (2012). Prevalence of metabolic syndrome in patients with psoriasis A
population-based study in the United Kingdom. J Invest Dermatol.

[22] Mehta NN1, Azfar RS, Shin DB, Neimann AL, Troxel AB, Gelfand JM (2010). Patients with severe psoriasis are at increased risk of
cardiovascular mortality cohort study using the General Practice Research
Database. Eur Heart J.

[23] Federman DG, Shelling M, Prodanovich S, Gunderson CG, Kirsner RS (2009). Psoriasis an opportunity to identify cardiovascular
risk. Br J Dermatol.

[24] Brauchli YB, Jick SS, Meier CR (2008). Psoriasis and the risk of incident diabetes mellitus: a
population-based study. Br J Dermatol.

[25] Mallbris L, Akre O, Granath F, Yin L, Lindelöf B, Ekbom A (2004). Increased risk for cardiovascular mortality in psoriasis
inpatients but not in outpatients. Eur J Epidemiol.

[26] Mallbris L, Granath F, Hamsten A, Ståhle M (2006). Psoriasis is associated with lipid abnormalities at the onset of
skin disease. J Am Acad Dermatol.

[27] Karadag AS, Yavuz B, Ertugrul DT, Akin KO, Yalcin AA, Deveci OS (2010). Is psoriasis a pre-atherosclerotic disease Increased insulin
resistance and impaired endothelial function in patients with
psoriasis. Int J Dermatol.

[28] Antoniou C, Dessinioti C, Katsambas A, Stratigos AJ (2007). Elevated triglyceride and cholesterol levels after intravenous
antitumour necrosis factor-alpha therapy in a patient with psoriatic
arthritis and psoriasis vulgaris. Br J Dermatol.

[29] Stinco G, Piccirillo F, Patrone P (2007). Hypertriglyceridaemia during treatment with adalimumab in
psoriatic arthritis. Br J Dermatol.

[30] Gus I, Fischmann A, Medina C (2002). Prevalência dos fatores de risco da doença arterial
coronariana no estado do Rio Grande do Sul. Arq Bras Cardiol.

[31] Brasil, Ministério da Saude, Instituto Nacional de Câncer, Coordenação de Prevenção e
Vigilância, Prevalência de Tabagismo no Brasil (2004). Dados dos Inquéritos Epidemiológicos em Capitais
Brasileiras.

[32] Souza LJ, Souto Filho JTD, Souza TF, Reis AFF, Gicovate Neto C, Bastos DA (2003). Prevalência de dislipidemia e fatores de risco em Campos
dos Goytacazes, RJ. Arq Bras Endocrinol Metab.

[33] Gisondi P, Girolomoni G (2009). Psoriasis and atherothrombotic diseases diseasespecific and
non-disease-specific risk factors. Semin Thromb Hemost.

[34] Papp K, Poulin Y, Vieira A (2013). Examining the risk of cardiovascular disease in patients with
psoriasis a critical review. J Cutan Med Surg.

[35] Gulliver W (2008). Long-term prognosis in patients with psoriasis. Br J Dermatol.

[36] Qureshi AA, Choi HK, Setty AR, Curhan GC (2009). Psoriasis and the risk of diabetes and hypertension a prospective
study of US female nurses. Arch Dermatol.

[37] Parsi KK, Brezinski EA, Lin TC, Li CS, Armstrong AW (2012). Are patients with psoriasis being screened for cardiovascular
risk factors A study of screening practices and awareness among primary care
physicians and cardiologists. J Am Acad Dermatol.

[38] Ahlehoff O, Skov L, Gislason G, Lindhardsen J, Kristensen SL, Iversen L (2012). Pharmacological undertreatment of coronary risk factors in
patients with psoriasis observational study of the Danish nationwide
registries. PLoS One.

[39] Vena GA, Vestita M, Cassano N (2010). Psoriasis and cardiovascular disease. Dermatol Ther.

[40] Naldi L, Gambini D (2007). The clinical spectrum of psoriasis. Clin Dermatol.

